# Neural integration of musical and lyrical emotion: semantic–acoustic congruence in song processing

**DOI:** 10.3389/fpsyg.2026.1973585

**Published:** 2026-10-27

**Authors:** Shuo Yang, Zhihan Wang

**Affiliations:** Herzen State Pedagogical University of Russia, Saint Petersburg, Russia

**Keywords:** affective neuroscience, EEG/ERP, emotional congruence, music and lyrics, neuroimaging, semantic–acoustic integration, song processing, vocal prosody

## Abstract

Songs convey emotion through the joint contribution of musical-acoustic structure, lyrical semantics, and vocal-prosodic expression, yet these information sources have often been examined separately in affective neuroscience. This Mini Review synthesizes behavioral, electroencephalographic/event-related potential (EEG/ERP), magnetoencephalographic (MEG), and functional neuroimaging evidence relevant to semantic–acoustic emotional congruence during song processing. Available findings suggest that lyrics and melodies engage partially dissociable but interacting processes during song perception. Early MEG responses around 130–140 ms differentiate lyric and melodic expectancy violations in memorized songs, while N400 modulations provide evidence of context-sensitive meaning-related processing across musical and verbal materials. Later positive components may reflect additional attentional allocation and evaluative processing under conflicting or ambiguous conditions, although their functional interpretations remain task-dependent. Functional neuroimaging studies have implicated superior temporal, middle temporal, and inferior frontal regions in lyric–melody processing, whereas studies of music-evoked emotion more broadly have reported activity in the amygdala, insula, cingulate cortex, hippocampus, and striatal regions. The specific contributions of these regions to music–lyric emotional congruence, however, remain uncertain. Behavioral evidence further suggests that congruence does not uniformly facilitate emotional processing and that incongruence may sometimes increase arousal or alter the relative weighting of musical and lyrical cues. On this basis, the review proposes a provisional dynamic weighted-integration account in which musical-acoustic affect, lyrical meaning, and vocal prosody interact across temporally overlapping processing stages, with their relative contributions varying according to expectations, attention, expertise, and language accessibility. Direct neural evidence specifically manipulating music–lyric emotional congruence remains limited, identifying an important direction for future naturalistic and multimodal research.

## 1 Introduction

### 1.1 From isolated music and language processing to song integration

Everyday song perception is intrinsically integrative. Unlike instrumental music or spoken language, songs simultaneously convey affect through musical-acoustic structure and linguistic meaning, while the singing voice binds these information streams within a single auditory event. Nevertheless, the neural study of emotion has often treated music and language separately: music research has emphasized acoustic and structural cues and their affective consequences, whereas language research has focused primarily on lexical-semantic and prosodic emotion (Brattico et al., 2011; Harris et al., 2023). This separation is increasingly difficult to sustain. Functional magnetic resonance imaging (fMRI) adaptation evidence suggests that lyrics and melodies have partially dissociable yet interacting representations within auditory cortical regions during song perception (Sammler et al., 2010). Complementing this finding, intracranial recordings and fMRI have identified a song-selective neural population in auditory cortex whose responses could not be explained by standard acoustic features (Norman-Haignere et al., 2022). Together, these findings suggest that song processing involves interactions between musical and linguistic information as well as auditory representations that are not readily reducible to low-level acoustic properties. Accordingly, studying melody or lyrics in isolation cannot fully explain emotional experience during natural song perception.

### 1.2 Semantic–acoustic emotional congruence

A central question is how the brain combines emotional information when these channels agree or conflict. A positively valenced musical context paired with positive lyrics constitutes *semantic–acoustic emotional congruence*, whereas, for example, a sad musical context combined with positive lyrics creates emotional incongruence. Such contrasts provide a tractable model for investigating cross-domain emotional integration because musical information can establish affective and semantic expectations that influence subsequent linguistic processing. Music–language priming studies have reported N400 modulations and behavioral differences between related and unrelated musical–verbal pairs, suggesting that musical context can influence subsequent meaning-related and evaluative processing (Koelsch et al., 2004; Steinbeis and Koelsch, 2011; Goerlich et al., 2012). However, these effects do not uniquely establish semantic integration, as affective priming may also involve response-related mechanisms, and the sequential presentation of musical primes and verbal targets differs from the simultaneous presentation of lyrics and melody in natural song. Moreover, congruence should not be equated with uniformly facilitated or intensified emotion. Behavioral studies of songs indicate that the relative weighting of melody and lyrics varies with emotional category and listener characteristics (Ali and Peynircioğlu, 2006; Vidas et al., 2020). Most recently, Karbanova and Cui (2026) found that music–lyrics congruence did not reliably predict valence ratings, whereas greater affective incongruence was associated with increased arousal. Emotional conflict may therefore sometimes heighten arousal rather than simply impose processing costs.

### 1.3 Aim and contribution

This Mini Review synthesizes behavioral, EEG/ERP, MEG, ECoG, and functional neuroimaging evidence to characterize the temporal and spatial characteristics of music–lyric interaction, distinguish direct findings from converging and indirect evidence, and develop a provisional semantic–acoustic emotion integration framework.

## 2 Literature search and scope

A focused literature search was conducted in PubMed, Web of Science Core Collection, Scopus, and APA PsycINFO to identify empirical studies examining music–lyric integration and emotional congruence during song perception. The searches were last updated on October 12, 2026. Search terms covered three conceptual domains: song-related information (song, lyrics, melody, singing, vocal music), emotional processing (emotion, affect, valence, arousal), and cross-domain relationships (congruence, incongruence, integration, interaction, conflict). To capture both behavioral and neural evidence, broad searches were supplemented by modality-specific searches incorporating terms related to electroencephalography (EEG), event-related potentials (ERP), magnetoencephalography (MEG), electrocorticography (ECoG), functional magnetic resonance imaging (fMRI), and neuroimaging. Neural-measure terms were not required in the broad searches, allowing relevant behavioral studies to be identified. Database-specific search strings, search fields, dates, and applied filters are provided in Supplementary Table S1.

Studies were considered eligible if they (a) reported original empirical findings involving human participants; (b) examined interactions between musical and lyrical information in songs or closely related music–language paradigms; and (c) provided behavioral or neural evidence relevant to semantic, affective, acoustic, or cross-domain processing. Particular attention was given to studies manipulating semantic, affective, melodic, or harmonic congruence. Studies involving isolated instrumental music or linguistic stimuli were included only when they directly informed the interpretation of music–language or affective integration. Non-empirical publications, animal studies, and investigations unrelated to music–language interactions or affective processing were excluded from the primary empirical synthesis. Review articles were used for theoretical context and reference identification rather than treated as primary empirical evidence.

The search covered publications from database inception to the final search date, without a publication-year restriction. Greater emphasis was placed on studies published between 2015 and 2026 when discussing recent developments, but earlier studies were not excluded. Foundational studies were retained when they provided original experimental demonstrations of influential paradigms or findings directly relevant to the review questions, such as early ERP evidence concerning lyrics and tunes (Besson et al., 1998) and fMRI adaptation evidence concerning lyric–melody representations (Sammler et al., 2010). Reference lists of relevant studies and reviews were also screened to identify additional publications. As a focused Mini Review, the synthesis was organized thematically to distinguish direct findings from converging and indirect evidence rather than to provide a quantitative meta-analysis. Throughout the review, “functional neuroimaging” refers primarily to hemodynamic imaging methods such as fMRI, whereas EEG/ERP, MEG, and ECoG findings are described separately by recording modality.

## 3 Semantic–acoustic emotion integration in songs

### 3.1 Acoustic, semantic, and vocal-prosodic sources of emotion

Emotional meaning in song arises from several partially dissociable information sources. The first is an acoustic-affective stream, in which musical properties such as mode, tempo, pitch contour, intensity, consonance, and timbre provide relatively direct cues to valence and arousal. In songs, however, acoustic affect is not restricted to accompaniment or melody. The singing voice itself carries emotional information through pitch variation, intensity, timing, spectral characteristics, and other prosodic cues. Functional neuroimaging studies of vocal emotion have reported activity in bilateral superior temporal regions, as well as inferior frontal and insular cortices, although the specific functional contributions of these regions depend on the experimental task and cannot be inferred from regional activation alone (Bestelmeyer et al., 2014; Schirmer, 2018).

A second source is the semantic-affective stream conveyed by lyrics. Lexical valence, sentence-level context, narrative meaning, and emotion concepts can generate affective expectations independently of how the words are sung. Thus, lyrical semantic emotion should not be equated with vocal emotion: the same positively valenced sentence can, in principle, be delivered with a sad or anxious vocal expression. Natural song perception therefore requires the brain to integrate musical acoustics, linguistic semantics, and vocal-prosodic affect rather than merely combine “music” and “lyrics”.

### 3.2 A dual-stream integration framework

Within this framework, vocal prosody is treated as a distinct affective cue source embedded primarily within the acoustic stream rather than as a third independent processing stream. Existing evidence supports a provisional dual-stream framework in which acoustic and linguistic-semantic information are partially dissociable but interact across temporally overlapping processing stages. Musical and vocal-acoustic information is rapidly processed in auditory cortical regions, including the superior temporal gyrus (STG) and superior temporal sulcus (STS), while affectively relevant differentiation can emerge during early auditory processing rather than only after a separate perceptual stage. For example, intracranial electrocorticographic (ECoG) recordings have revealed dissonance-sensitive high-gamma responses in the STG within approximately 75–200 ms (Foo et al., 2016), and EEG findings have identified early electrophysiological differentiation between affectively distinct chords (Kirsanov et al., 2025). In parallel, linguistic information engages auditory-phonological and lexical-semantic processes involving temporal and inferior frontal regions. Cortical and subcortical processes relevant to musical emotion may operate at overlapping temporal scales rather than within a strictly serial architecture (Koelsch, 2014). These pathways should therefore be viewed as partially differentiated but dynamically interacting, rather than as independent streams that converge only after acoustic analysis has been completed. Consistent with this view, fMRI adaptation findings indicate interactions between lyric and melodic representations in superior temporal regions during song perception (Sammler et al., 2010).

Two complementary temporal emphases can be distinguished. An early-interaction account emphasizes the influence of musical context on linguistic processing during relatively early perceptual and predictive stages, whereas a later-integration account emphasizes additional semantic evaluation and reappraisal when differentiated sources of information must be reconciled (Besson et al., 1998; Gordon et al., 2010; Rosslau et al., 2016). These accounts need not imply mutually exclusive or strictly sequential stages, as early perceptual differentiation and later evaluative processing may overlap in time.

At higher levels, lyric–melody interactions may involve inferior frontal and prefrontal regions, whereas studies of music-evoked emotion more broadly have reported activity in the amygdala, anterior insula, cingulate cortex, hippocampus, and striatum. However, these regional findings do not establish specific functional roles in music–lyric emotional congruence, particularly when the underlying studies did not directly manipulate emotional conflict or model salience, valuation, or reward-related processes. Accordingly, the proposed framework should be regarded as a testable synthesis rather than an established neural architecture. Acoustic, linguistic, and affective processes may interact across overlapping temporal stages, with additional evaluation and cue reconciliation occurring when musical and lyrical information converge or conflict.

## 4 Neural evidence for emotional congruence and conflict

### 4.1 Behavioral evidence: facilitation versus interference

Behavioral evidence indicates that congruence between musical and linguistic affect is psychologically consequential, although its effects are not uniformly facilitatory. Ali and Peynircioğlu (2006) showed that melodies generally exerted a stronger influence than lyrics on perceived song emotion, while the contribution of lyrics varied with emotion category: adding lyrics tended to strengthen negative emotions such as sadness and anger but attenuate positive emotions such as happiness and calmness. Developmental evidence further demonstrates that cue weighting is not fixed. In 172 participants aged 7–20 years, recognition of emotion in congruent popular songs improved with age, whereas judgments of incongruent songs shifted between lyrical and melodic information depending on both age and the specific emotion conveyed (Vidas et al., 2020). Related cross-modal priming evidence has also reported faster and more accurate responses under affectively congruent musical-prime and emotional-target conditions (Zhang et al., 2018), although such sequential priming effects do not directly establish the mechanisms of simultaneous music–lyric integration.

More recent findings complicate a simple facilitation account. Karbanova and Cui (2026) observed a 17-ms lexical-decision advantage for concepts associated with the musical rather than lyrical component of song primes. Music–lyric affective distance did not predict valence ratings, but greater incongruence predicted higher arousal (*p* = 0.030, marginal *R*^2^ = 0.188). Thus, congruence may facilitate categorization under some conditions, whereas incongruence may be associated with heightened arousal. Whether such effects reflect increased reweighting of competing emotional cues remains a question for future experimental research.

### 4.2 Temporal dynamics: EEG/ERP, MEG, and ECoG evidence

Time-resolved EEG/ERP, MEG, and ECoG findings suggest that musical-acoustic, linguistic, and affective information can be differentiated during early processing and may interact across temporally overlapping stages. In a MEG study of memorized songs, Yasui et al. (2009) reported a left-dominant M140 response to unexpected lyric substitutions and a right-dominant M130 response to unexpected melodic notes. The authors further distinguished these components from the magnetic mismatch negativity (MMN) elicited in a separate frequency-deviance condition, suggesting that the responses were associated with expectations concerning memorized lyrics and melodies rather than simple acoustic mismatch. Although these findings concern expectancy violations rather than emotional congruence, they provide study-specific evidence that lyric- and melody-related predictions can influence early neural responses.

At intermediate latencies, N400 effects provide evidence of context-sensitive processing involving musical and linguistic information, although their functional interpretation is not limited to semantic integration. Besson et al. (1998) reported relatively differentiated electrophysiological responses to semantic and musical violations in songs, whereas Gordon et al. (2010) demonstrated behavioral and ERP interactions between word and melody processing in sung material. Notably, Gordon et al. (2010) found that melodic differences could modulate N400 responses even when the linguistic target remained unchanged. Related music–language priming studies have also reported larger N400 responses to words that were conceptually unrelated rather than related to preceding musical excerpts (Koelsch et al., 2004; Daltrozzo and Schön, 2009a). These findings suggest that musical context can influence subsequent meaning-related processing, but they do not support a simple separation between early expectancy responses and a later, exclusively semantic N400.

The interpretation of affective priming effects requires additional caution. Steinbeis and Koelsch (2011) reported faster evaluations of emotional words when their affective valence matched that conveyed by preceding musical sounds, together with N400 modulations across manipulations of consonance, mode, and timbre. However, affective priming can arise from multiple mechanisms, including facilitated target processing, associative activation, and response-related compatibility or competition. Because participants explicitly evaluated target words in the experiments of Steinbeis and Koelsch (2011), response-level contributions cannot be ruled out. N400 effects are also sensitive to task demands: Daltrozzo and Schön (2009b) reported a smaller and later music-to-word N400 effect under lexical decision than under explicit conceptual-relatedness judgment. More generally, the extent to which N400 activity reflects predictive pre-activation, facilitated access, or post-access integration remains debated (Lau et al., 2008; Kutas and Federmeier, 2011). Thus, music–language priming findings are best interpreted as evidence that musical context can modulate subsequent meaning-related and evaluative processing, without uniquely identifying the underlying mechanism. Moreover, these paradigms present musical primes and linguistic targets sequentially, whereas lyrics and melody unfold simultaneously during natural song perception.

Later positive activity may reflect additional evaluation when affective information becomes more complex, although such interpretations remain paradigm-dependent. Zhang et al. (2018) found that affective incongruence following instrumental musical primes increased N400 amplitude at 250–450 ms, whereas incongruence following music containing lyrics primarily elicited a larger late positive complex (LPC) at 500–700 ms. Importantly, this study used emotional faces as targets rather than directly manipulating emotional conflict between lyrics and melodies. Similarly, late positive responses to melodic changes in sung material have been reported by Gordon et al. (2010), but these effects do not uniquely establish emotional reappraisal. Taken together, the available findings are compatible with early differentiation, context-sensitive semantic-affective processing, and additional later evaluation. However, these processes should not be interpreted as a universal sequence of discrete stages, and direct time-resolved evidence for neural processing of music–lyric emotional congruence remains limited.

### 4.3 Spatial organization: functional neuroimaging evidence

Functional neuroimaging studies provide evidence concerning cortical representations involved in lyric–melody processing, although the neural organization of emotional congruence between musical and lyrical information remains insufficiently established. Using an fMRI adaptation paradigm, Sammler et al. (2010) reported partially dissociable yet interacting responses to lyrics and tunes across bilateral superior temporal regions. These findings provide evidence concerning lyric–melody representations during unfamiliar-song perception but do not independently establish the temporal stages or affective functions of their interaction.

Using a subsequent-memory paradigm, Alonso et al. (2016) investigated neural activity associated with encoding new songs under two presentation conditions. In the unified condition, melodies were sung with lyrics, whereas in the separate condition, melodies were sung on the neutral syllable “la” while lyrics were presented visually. Written lyrics were displayed in both conditions, and participants were instructed to memorize the material. Successful binding in the unified condition was associated with activity in the left inferior frontal gyrus (IFG), bilateral middle temporal gyrus (MTG), and left motor cortex, whereas the separate condition was associated with greater activity in the right hippocampus, left caudate, cerebellum, and right IFG. These findings identify regions involved in binding verbal and melodic information under specific encoding and memory demands. However, because the paradigm involved explicit memorization and differed in the sensory presentation and integration demands of its conditions, these findings should not be generalized to an established neural architecture for lyric–melody integration during naturalistic song perception.

Affective neuroimaging provides a related but distinct source of evidence. Brattico et al. (2011) reported that neural responses to music with and without lyrics varied according to the emotional character of the musical material. For sad music, the presence of lyrics was associated with differences in auditory and inferior frontal regions as well as the amygdala, parahippocampal gyrus, and putamen. Other contrasts involving happy music showed activity differences in regions including the anterior cingulate cortex (ACC) and insula. More broadly, an activation likelihood estimation (ALE) meta-analysis of 40 neuroimaging studies identified the amygdala, insula, striatum, hippocampus, ACC, and STG among regions associated with music-evoked emotional processing (Fuentes-Sánchez et al., 2025). Importantly, this meta-analysis excluded music containing lyrics. These findings therefore provide contextual evidence concerning neural responses to musical emotion rather than direct evidence for music–lyric emotional congruence.

The functional interpretation of these regional findings requires caution. Although the ACC, lateral prefrontal cortex, amygdala, insula, and striatum may represent candidate regions for future investigation of conflict-related and affective processes, activity in these multifunctional regions does not establish any specific psychological computation. Inferring emotional salience, valuation, reward, or conflict monitoring solely from regional activation would risk reverse inference (Poldrack, 2006). Furthermore, activation-based findings do not establish functional connectivity or coordinated activity among these regions. Direct neuroimaging studies that systematically contrast emotionally congruent and incongruent musical–lyrical combinations are therefore needed before claims concerning dedicated conflict-related circuits, reward-related mechanisms, or connectivity changes can be substantiated.

### 4.4 What is established—and what remains inferential?

Three levels of evidence can currently be distinguished. First, individual behavioral, EEG/ERP, MEG, and fMRI studies provide evidence that lyrics and melodies engage partially dissociable but interacting processes under specific experimental conditions, including expectancy violations, song perception, and memory encoding. However, the extent to which these findings generalize across naturalistic song-listening contexts remains uncertain. Second, emerging behavioral findings suggest that affective congruence and incongruence can influence evaluative responses and arousal, whereas changes in cue weighting and neural integration demands remain more tentative interpretations. Still inferential, however, are the specific neural computations underlying emotional conflict. Current evidence is insufficient to conclude that incongruent songs selectively recruit an ACC–dlPFC conflict network, that congruence strengthens STG–striatal connectivity, or that matched musical and lyrical emotions generate superadditive reward responses. This evidential asymmetry is theoretically informative: existing studies have characterized several component processes involved in song perception, but the neural computations specifically supporting emotional agreement and conflict between lyrics and music remain largely unresolved.

## 5 Moderators of music–lyric integration

### 5.1 Musical expertise

Musical expertise may alter the relative weighting of acoustic and linguistic information without producing a fixed “music-dominant” processing strategy. Long-term training is associated with enhanced auditory encoding, greater sensitivity to melodic expectancy, and stronger top-down modulation of auditory cortical responses (Strait and Kraus, 2014; Park et al., 2018). In song-specific MEG evidence, professional singers detected pitch violations more accurately than actors and showed greater late activity in right temporal and left parietal regions, although early neural responses to semantic and melodic violations did not differ reliably between groups (Rosslau et al., 2016). These findings suggest that musical expertise may influence the processing of melodic and linguistic information in song, although direct evidence that training systematically changes the relative weighting of musical and lyrical emotion remains limited.

### 5.2 Language familiarity

Language familiarity may influence the accessibility of lyrical meaning and consequently alter the relative contributions of musical-acoustic, semantic, and vocal-prosodic information during song perception. When listeners have limited understanding of the language being sung, access to lexical-semantic content is reduced, whereas musical structure and vocal-prosodic cues remain available. Cross-cultural behavioral evidence suggests that the contribution of lyrics to music-evoked emotion varies according to listeners’ linguistic and cultural backgrounds (Barradas and Sakka, 2022). Related research on unfamiliar-language emotional prosody further suggests that some vocal-affective information can be perceived without full comprehension of lexical content (Birse et al., 2026). In addition, recent EEG evidence indicates that linguistic expectations can influence auditory processing during song listening, including P200 and gamma-band responses associated with perceived prediction violations (Giraldi et al., 2026). These findings suggest that linguistic accessibility and prior expectations may modulate song perception, although their precise contributions to emotional congruence remain to be established.

Songs performed in unfamiliar languages provide a particularly informative context for distinguishing acoustic-affective information from lyrical semantics. When listeners cannot understand the lyrics, semantic access is substantially restricted while musical and vocal cues remain available. Cross-cultural work has shown that listeners can infer the behavioral functions of songs from unfamiliar societies on the basis of their musical form (Mehr et al., 2018), and that acoustic features can predict the behavioral contexts of songs across societies (Mehr et al., 2019). Although these findings do not directly measure neural integration of lyrical emotion, they suggest that musical-acoustic information can support meaningful judgments independently of familiar lexical-semantic content. Direct neural comparisons between songs with comprehended and non-comprehended lyrics are therefore needed to determine how semantic accessibility changes the temporal and spatial characteristics of music–lyric integration. Language familiarity should consequently be conceptualized as a continuous moderator of semantic access rather than a categorical distinction between linguistic and purely musical listening.

### 5.3 Attention and task demands

The weighting of lyrics and melody also depends on what listeners are instructed to attend to. Gordon et al. (2010) directly manipulated attention by asking listeners to judge either words or melodies in sung stimuli. Word changes elicited an N400 in both tasks, indicating that lexical-semantic information continued to be processed even when attention was directed toward melody; however, the N400 word effect was smaller during the musical task. Melodic changes likewise influenced electrophysiological responses even when melody was not the explicit task target, and word–melody interactions remained detectable across attentional conditions. Attention therefore appears to modulate rather than completely gate cross-domain processing. Consequently, although direct evidence concerning attentional modulation of music–lyric emotional congruence remains limited, related findings suggest that future studies should consider attentional set, judgment criteria, and processing goals as potential moderators.

## 6 Discussion and future directions

### 6.1 Toward a dynamic integration account

The evidence reviewed here argues against treating song emotion as the additive result of “music emotion + lyric emotion.” A provisional alternative is a dynamic weighted-integration account, in which musical-acoustic, lyrical-semantic, and vocal-prosodic cues contribute jointly to emotional interpretation. Within this proposed account, the relative influence of musical-acoustic affect, lyrical-semantic meaning, and vocal-prosodic expression may vary dynamically according to cue reliability, prior expectations, language accessibility, expertise, and attentional goals. Congruent cues may facilitate coherent emotional interpretation, whereas incongruent cues may prompt additional evaluation or changes in cue weighting. These mechanisms are proposed theoretical processes rather than computations directly established by existing music–lyric congruence studies. Incongruent cues, however, need not simply impair processing. They may trigger reweighting of the available cues, conflict monitoring, and later reappraisal. Consistent with this view, recent behavioral evidence indicates that greater affective distance between music and lyrics can be associated with increased arousal even when it does not systematically alter valence judgments (Karbanova and Cui, 2026). Thus, incongruence may sometimes heighten arousal and promote additional cue weighting or reappraisal, rather than function solely as a processing cost. The framework proposed in Figure 1 therefore conceptualizes congruence as a dynamic relation among potentially competing affective cues and predictions, rather than as a fixed property that uniformly enhances emotional responses.

**Figure 1:**
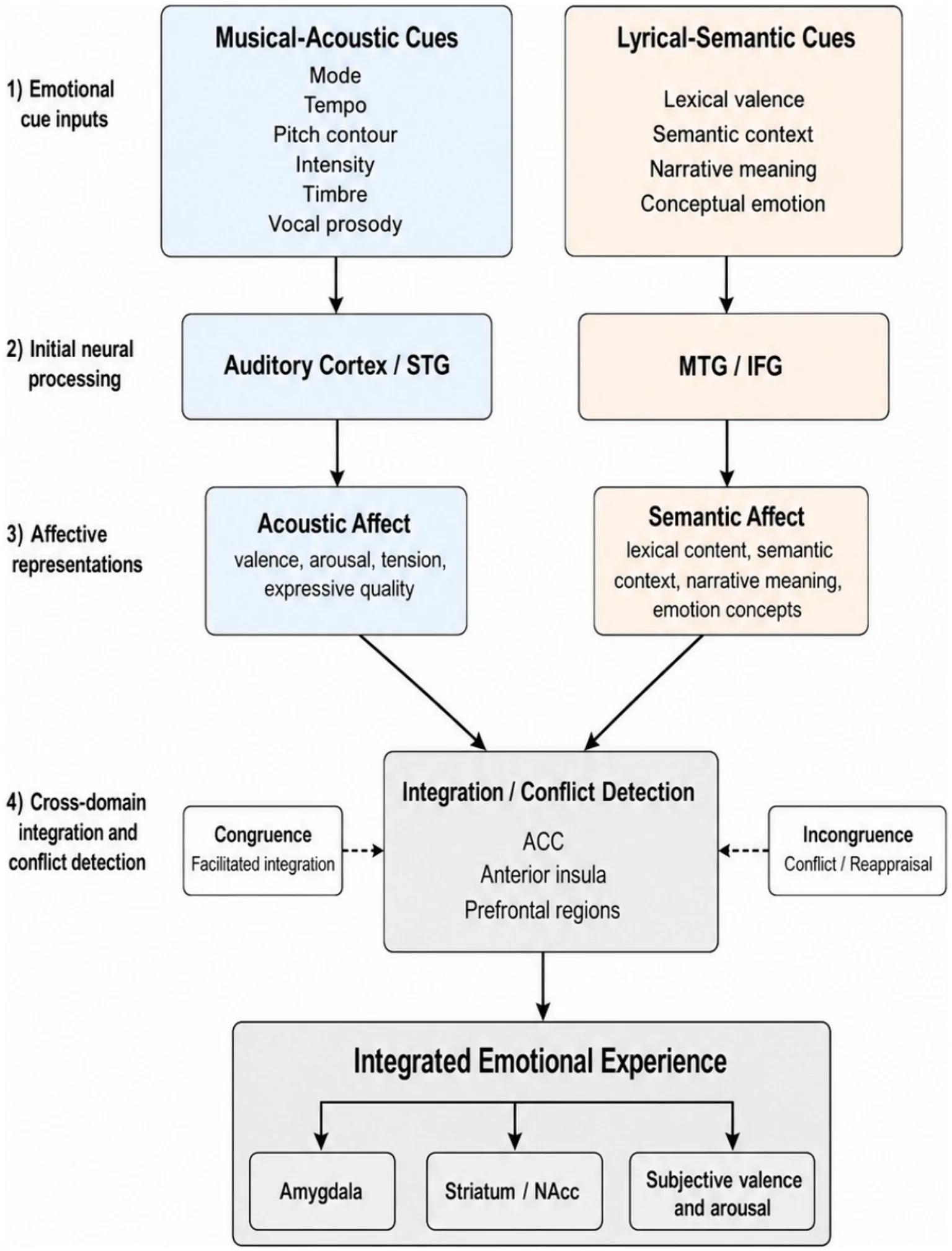
A dual-stream model of semantic–acoustic emotion integration during song processing.

### 6.2 Methodological limitations

Three limitations constrain current conclusions. First, ecological validity remains uneven. Many EEG and neuroimaging paradigms rely on brief, simplified, or experimentally altered musical materials that improve control but remove the temporal development, expressive timing, and contextual meaning characteristic of real songs (Tervaniemi, 2023). Second, studies often insufficiently separate lyrical semantics from vocal-prosodic emotion. A sentence with positive lexical meaning can be sung with sad, tense, or ironic prosody, and affective prosody itself recruits partly overlapping frontotemporal and subcortical systems involved in emotional evaluation (Themistocleous, 2025). Consequently, an apparent “music–lyrics” congruence effect may actually reflect an unresolved interaction among musical affect, semantic affect, and vocal-prosodic affect. Without orthogonal manipulation of these sources, their independent neural contributions cannot be identified confidently. Third, the existing literature is heterogeneous in experimental design, behavioral task, and neural recording modality. Several influential findings originate from individual studies using memorized songs, subsequent-memory tasks, or sequential music–language priming paradigms rather than direct manipulations of emotional congruence in naturalistic song listening. These differences limit the generalizability of individual findings and complicate the integration of evidence across studies. Moreover, regional activation findings alone cannot identify specific psychological computations or establish functional connectivity between brain regions. Accordingly, the proposed framework should be evaluated through studies that directly manipulate music–lyric emotional congruence while minimizing confounds related to task demands, language comprehension, and stimulus presentation.

### 6.3 Future research agenda

Future work should combine experimental control with naturalistic song listening. Full-length, professionally performed songs can be paired with continuous valence/arousal ratings and intersubject correlation or dynamic functional-connectivity analyses to identify moments at which listeners’ neural responses converge or networks reconfigure; such approaches have already proved informative for naturalistic music-evoked emotion (Trost et al., 2015; Sachs et al., 2020). EEG/MEG and fMRI could further provide complementary temporal and spatial constraints, while representational-similarity analyses may test whether acoustic, semantic, and affective representations become more similar across processing stages. Most critically, future studies should orthogonally manipulate musical emotion (happy/sad) × lyrical-semantic emotion (happy/sad) × vocal prosody (happy/sad) in a 2 × 2 × 2 factorial design. This would separate the three affective information sources and directly test when, where, and under what conditions semantic–acoustic congruence becomes neurally consequential. An additional priority is to compare songs presented in familiar and unfamiliar languages while holding musical structure and vocal delivery as constant as possible. Such designs could test whether reduced semantic accessibility changes behavioral evaluations, early neural responses, or later cross-domain interactions. Combining language-comprehension manipulations with temporally resolved neural measures would help distinguish the contributions of lyrical meaning, musical-acoustic affect, and vocal prosody without assuming a strictly serial processing architecture.

## 7 Conclusion

Song emotion cannot be fully explained by considering melody, lyrics, and vocal expression in isolation. Available behavioral, EEG/ERP, MEG, ECoG, and functional neuroimaging findings suggest that musical-acoustic and linguistic-semantic information engage partially dissociable yet interacting processes, with affectively relevant information potentially emerging across temporally overlapping stages. However, much of the existing evidence derives from specific expectancy, priming, and memory-encoding paradigms rather than direct neural investigations of emotional congruence between music and lyrics. Consequently, the proposed dynamic weighted-integration account should be regarded as a provisional framework rather than an established neural mechanism. Future research combining direct manipulations of musical, lyrical, and vocal-prosodic emotion with naturalistic listening and multimodal neural recording may clarify how convergent and conflicting affective cues contribute to emotional experience during song perception.
