# Supplementary material for: Neural integration of musical and lyrical emotion: semantic–acoustic congruence in song processing
Source: Front Psychol. 2026 Oct 27;17:1973585. doi: 10.3389/fpsyg.2026.1973585 (PMC13749167; doi:10.3389/fpsyg.2026.1973585)
Supplement: Supplementary file 1 [file Supplementary_file_1.DOCX]

Supplementary Table S1. Database-specific literature search strategies for studies of music–lyric integration and emotional congruence

| **Database** | **Search fields** | **Search strategy** | **Search date** | **Filters** | **Records retrieved** |
| --- | --- | --- | --- | --- | --- |
| PubMed | Title/Abstract | S1: Broad search | 12 Oct 2026 | English; Humans | 286 |
| PubMed | Title/Abstract | S2: Neural-specific search | 12 Oct 2026 | English; Humans | 94 |
| Web of Science Core Collection | Topic | S1: Broad search | 12 Oct 2026 | English; Article | 524 |
| Web of Science Core Collection | Topic | S2: Neural-specific search | 12 Oct 2026 | English; Article | 178 |
| Scopus | Title/Abstract/Keywords | S1: Broad search | 12 Oct 2026 | English; Article | 618 |
| Scopus | Title/Abstract/Keywords | S2: Neural-specific search | 12 Oct 2026 | English; Article | 203 |
| APA PsycINFO (EBSCOhost) | Title/Abstract | S1: Broad search | 12 Oct 2026 | English; Peer reviewed | 241 |
| APA PsycINFO (EBSCOhost) | Title/Abstract | S2: Neural-specific search | 12 Oct 2026 | English; Peer reviewed | 76 |

**Exact search strings**

**A. PubMed**

S1. Broad search

(song[tiab] OR songs[tiab] OR lyric*[tiab]

OR melod*[tiab] OR singing[tiab]

OR "vocal music"[tiab] OR "sung words"[tiab])

AND

(

emotion*[tiab] OR affect*[tiab]

OR valence[tiab] OR arousal[tiab]

OR congruen*[tiab] OR incongruen*[tiab]

OR integrat*[tiab] OR interact*[tiab]

OR conflict*[tiab] OR semantic*[tiab]

OR processing[tiab] OR binding[tiab]

OR priming[tiab] OR expectancy[tiab]

)

S2. Neural-specific search

(song[tiab] OR songs[tiab] OR lyric*[tiab]

OR melod*[tiab] OR singing[tiab]

OR "vocal music"[tiab] OR "sung words"[tiab])

AND

(EEG[tiab] OR ERP[tiab] OR MEG[tiab]

OR ECoG[tiab] OR fMRI[tiab]

OR neuroimag*[tiab]

OR electrophysiolog*[tiab]

OR "event-related potential"[tiab]

OR "event-related potentials"[tiab]

OR magnetoencephalograph*[tiab]

OR electroencephalograph*[tiab]

OR electrocorticograph*[tiab])

AND

(emotion*[tiab] OR affect*[tiab]

OR semantic*[tiab] OR lyric*[tiab]

OR integrat*[tiab] OR interact*[tiab]

OR processing[tiab] OR expectancy[tiab])

**B. Web of Science Core Collection**

S1. Broad search

TS=(song OR songs OR lyric* OR melod*

OR singing OR "vocal music" OR "sung words")

AND

TS=(emotion* OR affect* OR valence OR arousal

OR congruen* OR incongruen* OR integrat*

OR interact* OR conflict* OR semantic*

OR processing OR binding OR priming OR expectancy)

S2. Neural-specific search

TS=(song OR songs OR lyric* OR melod*

OR singing OR "vocal music" OR "sung words")

AND

TS=(EEG OR ERP OR MEG OR ECoG OR fMRI

OR neuroimag* OR electrophysiolog*

OR "event-related potential*"

OR magnetoencephalograph*

OR electroencephalograph*

OR electrocorticograph*)

AND

TS=(emotion* OR affect* OR semantic*

OR lyric* OR integrat* OR interact*

OR processing OR expectancy)

**C. Scopus**

S1. Broad search

TITLE-ABS-KEY(

song OR songs OR lyric* OR melod*

OR singing OR "vocal music" OR "sung words"

)

AND

TITLE-ABS-KEY(

emotion* OR affect* OR valence OR arousal

OR congruen* OR incongruen*

OR integrat* OR interact* OR conflict*

OR semantic* OR processing OR binding

OR priming OR expectancy

)

S2. Neural-specific search

TITLE-ABS-KEY(

song OR songs OR lyric* OR melod*

OR singing OR "vocal music" OR "sung words"

)

AND

TITLE-ABS-KEY(

EEG OR ERP OR MEG OR ECoG OR fMRI

OR neuroimag* OR electrophysiolog*

OR "event-related potential*"

OR magnetoencephalograph*

OR electroencephalograph*

OR electrocorticograph*

)

AND

TITLE-ABS-KEY(

emotion* OR affect* OR semantic*

OR lyric* OR integrat* OR interact*

OR processing OR expectancy

)

**D. APA PsycINFO (EBSCOhost)**

S1. Broad search

(

TI (song OR songs OR lyric* OR melod*

OR singing OR "vocal music" OR "sung words")

OR

AB (song OR songs OR lyric* OR melod*

OR singing OR "vocal music" OR "sung words")

)

AND

(

TI (emotion* OR affect* OR valence OR arousal

OR congruen* OR incongruen* OR integrat*

OR interact* OR conflict* OR semantic*

OR processing OR binding OR priming OR expectancy)

OR

AB (emotion* OR affect* OR valence OR arousal

OR congruen* OR incongruen* OR integrat*

OR interact* OR conflict* OR semantic*

OR processing OR binding OR priming OR expectancy)

)

S2. Neural-specific search

(

TI (song OR songs OR lyric* OR melod*

OR singing OR "vocal music" OR "sung words")

OR

AB (song OR songs OR lyric* OR melod*

OR singing OR "vocal music" OR "sung words")

)

AND

(

TI (EEG OR ERP OR MEG OR ECoG OR fMRI

OR neuroimag* OR electrophysiolog*

OR "event-related potential*"

OR magnetoencephalograph*

OR electroencephalograph*

OR electrocorticograph*)

OR

AB (EEG OR ERP OR MEG OR ECoG OR fMRI

OR neuroimag* OR electrophysiolog*

OR "event-related potential*"

OR magnetoencephalograph*

OR electroencephalograph*

OR electrocorticograph*)

)

AND

(

TI (emotion* OR affect* OR semantic* OR lyric*

OR integrat* OR interact* OR processing

OR expectancy)

OR

AB (emotion* OR affect* OR semantic* OR lyric*

OR integrat* OR interact* OR processing

OR expectancy)

)
